# Size-Dependent Oxidation-Induced Phase Engineering for MOFs Derivatives Via Spatial Confinement Strategy Toward Enhanced Microwave Absorption

**DOI:** 10.1007/s40820-022-00841-5

**Published:** 2022-04-12

**Authors:** Hanxiao Xu, Guozheng Zhang, Yi Wang, Mingqiang Ning, Bo Ouyang, Yang Zhao, Ying Huang, Panbo Liu

**Affiliations:** 1 School of Chemistry and Chemical Engineering, μNorthwestern Polytechnical University, Xi’an, 710129 People’s Republic of China; 2 Key Laboratory of Magnetic Materials and Devices, Ningbo Institute of Materials Technology & Engineering, Chinese Academy of Sciences, Ningbo, 315201 People’s Republic of China; 3 MIIT Key Laboratory of Semiconductor Microstructure and Quantum Sensing, Nanjing University of Science and Technology, Nanjing, 210094 People’s Republic of China; 4 Department of Mechanical and Materials Engineering, University of Western Ontario, London, ON N6A 5B9 Canada

**Keywords:** Size-dependent oxidation, Phase engineering, Coherent interface, Dielectric polarization, Electron holography

## Abstract

Precisely reducing the size of metal-organic frameworks (MOFs) derivatives is an effective strategy to manipulate their phase engineering owing to size-dependent oxidation; however, the underlying relationship between the size of derivatives and phase engineering has not been clarified so far. Herein, a spatial confined growth strategy is proposed to encapsulate small-size MOFs derivatives into hollow carbon nanocages. It realizes that the hollow cavity shows a significant spatial confinement effect on the size of confined MOFs crystals and subsequently affects the dielectric polarization due to the phase hybridization with tunable coherent interfaces and heterojunctions owing to size-dependent oxidation motion, yielding to satisfied microwave attenuation with an optimal reflection loss of −50.6 dB and effective bandwidth of 6.6 GHz. Meanwhile, the effect of phase hybridization on dielectric polarization is deeply visualized, and the simulated calculation and electron holograms demonstrate that dielectric polarization is shown to be dominant dissipation mechanism in determining microwave absorption. This spatial confined growth strategy provides a versatile methodology for manipulating the size of MOFs derivatives and the understanding of size-dependent oxidation-induced phase hybridization offers a precise inspiration in optimizing dielectric polarization and microwave attenuation in theory.

**Supplementary Information:**

The online version contains supplementary material available at 10.1007/s40820-022-00841-5.

## Highlights

- The size of metal organic frameworks (MOFs) derivatives was manipulated by a spatial confined growth strategy.
- Dielectric polarization is the dominant dissipation mechanism due to the phase hybridization based on size dependent oxidation motion.
- The specific reflection loss of synthesized Co/Co_3_O_4_ hollow carbon nanocages surpasses most reported MOFs derived counterparts for practical microwave absorption applications.

**Supplementary Information:**

The online version contains supplementary material available at 10.1007/s40820-022-00841-5.

## Graphical abstract

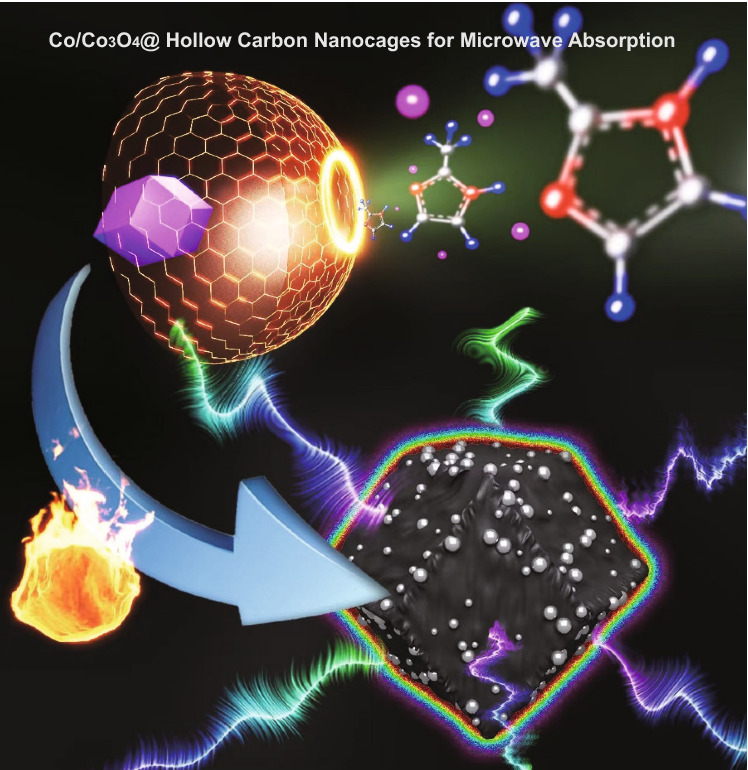

## Introduction

With the ever-increasing demand of electronic safety defense technology, smart microwave absorption devices with flexible characteristics, lightweight, ultrathin thickness, and high efficient performance are significantly pursued and promoted in civil and military electronic instruments [1, 2]. In recent decades, tremendous efforts have been attempted to develop functional absorbers to solve the serious electromagnetic radiation pollution; therefore, many promising candidates, such as carbon nanotubes [3], graphene [4–6], Mxene [7], and metal oxide/sulfide [8, 9], have attracted considerable attention, especially for their hybrid composites with multiple magnetic–dielectric components. In this case, optimized electromagnetic parameters and strong attenuation capability can be simultaneously achieved by manipulating the chemical components. In view of the advantage of morphologies (shape and size), several morphological-dependency investigations have manifested that structural engineering is also regarded as an effective strategy for tailoring the impedance characteristics, boosting microwave consumption and the most important characteristic, reducing the loading content. Recently, significant efforts have been devoted to constructing hierarchical absorbers, including multilayer core–shell or yolk–shell structures [10–12], hollow spheres [13, 14], or 3D porous foams [15–17], to reduce the density and optimize the impedance matching, which can trigger interfacial polarization and induce multiple reflection loss. Benefiting from the synergistic effect of structural merits and multiple loss coordination, the novel nanostructures with porous characteristics and large heterogeneous interfaces can simultaneously satisfy microwave consumption and decrease loading content to meet the requirement of high efficient absorption.

Nowadays, numerous scientific studies have demonstrated that metal–organic frameworks (MOFs)-derived strategy is one of the emerging research fields in the application of microwave absorption [18–23]. However, we get insight that changing microstructures and adjusting chemical components are still the two mainstreams to tune the microwave absorption for MOFs derivatives [24–26], in which dielectric polarization originated from the smaller size has been restricted more or less due to large size derivatives with unsatisfied heterogeneous junctions. The effect of phase hybridization, owing to size-dependent oxidation, on the microwave attenuation for MOFs derivatives has rarely been reported. Besides, it is well recognized that most MOFs derivatives exhibit solid morphology, high loading content, and impedance mismatch [27]. To address the dielectric polarization issue, a popular strategy is to reduce the size of MOFs derivatives to nanoscale because smaller size will endow the derivatives with large specific surface area, triggering enhanced dielectric polarization. Moreover, it is generally accepted that smaller derivatives produce more unavoidable defects and active sites, leading to phase hybridization due to the size-dependent oxidation, thus inducing interfacial polarization between the heterojunctions and coherent interfaces [28, 29], but the underlying mechanism between phase hybridization and microwave attenuation is based on semiempirical rules because these reduced nanoparticles are tend to aggregate or protected by carbon layers, in which the phase hybridization owning to size-dependent oxidation motion is prohibited. With regard to solid morphology for MOFs derivatives, it has been widely recognized that hollow engineering is an effective strategy to decrease the loading content. Hence, classical sacrificing templates [30], solvent etching [31], or synergistic protecting–etching strategy [32] have been used to construct hollow MOFs derivatives, which can indeed solve the loading content issue and manipulate the impedance characteristics, but the large inner hollow cavity limits the interfacial polarization to some degree. Therefore, precisely reduce the size of MOFs derivatives with respect to manipulate their phase hybridization and strengthen dielectric polarization, and simultaneously construct hollow cavity so as to meet the requirement of lightweight characteristics are highly desirable, but still face the bottlenecks and huge challenges.

Herein, we propose an internal growth strategy to confine the size of Co-based zeolitic imidazolate (ZIF-67) crystals, in which geometrically confined Co/Co_3_O_4_ derivatives, owing to the size-dependent oxidation, are encapsulated into hollow carbon nanocages (HCNs). It is believed that dielectric polarization increases due to phase hybridization with coherent interfaces, heterojunctions, and hierarchical pores, which are characterized by the simulated calculation and Lorentz off-axis electron hologram. The dielectric HCNs shell with internal hollow cavity effectively overcomes the shortcoming of high loading content, favors conduction loss, and optimizes matching impedance. The Co/Co_3_O_4_@HCNs absorbers exhibit an optimal reflection loss of −50.6 dB and an impressive bandwidth of 6.6 GHz with 20 wt% loading content, and the high specific reflection loss surpasses most MOFs-derived counterparts.

## Experimental and Calculation

### Synthesis of Hollow Carbon Nanocages

Resorcinol–formaldehyde (RF) resin was used as carbon source, and SiO_2_ spheres were used as sacrificing template to synthesize HCNs. In a typical procedure, 6 mL of TEOS and 2 mL ammonia (28 wt%) were firstly dissolved in 60 mL H_2_O/ethanol solvent (v/v = 1:1) and stirred for 30 min. After that, 0.4 g resorcinol and 0.6 mL formaldehyde were added, and the mixture solution was stirred for 24 h. The precipitates were collected by centrifugation with deionized water and dried at 60 °C for 12 h. After annealing at 800 °C for 4 h under N_2_ atmosphere, the products were washed with NaOH solution (1 mol L^−1^) at 65 °C for 10 h to completely etch SiO_2_ spheres.

### Synthesis of ZIF-67@HCNs

0.01 g HCNs was dispersed in 2.5 mL methanol and stirred for 30 min. After that, 0.0291 g Co(NO_3_)_2_·6H_2_O and 0.0328 g 2-methylimidazole were added and stirred for 25 min. The obtained ZIF-67@HCNs were collected by centrifugation.

### Synthesis of Co/Co_3_O_4_@HCNs

The as-prepared ZIF-67@HCNs were annealed at 800 °C for 2 h under N_2_ atmosphere, in which the ZIF-67 precursors were in situ transformed into dissociative Co/Co_3_O_4_ due to the size-dependent oxidation.

### Characterization

The morphologies were observed by field emission scanning electron microscopy (FESEM, Verios G4) and transmission electron microscopy (TEM, FEI Talos F200X TEM). The chemical compositions were characterized via X-ray diffractometer (XRD, Bruker, D8 DISCOVER A25). The Fourier-transform infrared (FTIR) spectra were measured by FTIR spectrophotometer (Varian 7000). The Raman spectroscopy was measured by WITec Alpha300R. The surface composition and valence state of elements were analyzed by XPS (Phoibos 100 spectrometer). The N_2_ adsorption–desorption isotherms and pore-size distribution were obtained on a pore structure and specific surface area analyzer (Micromeritics ASAP2460). The static magnetic properties were analyzed by multifunctional physical property measurement system (PPMS, CFMS-14 T). The samples were dispersed in paraffin matrix with 20 wt%, which were made into a circular ring with an internal diameter of 3.0 mm and external diameter of 7.0 mm. The minimum reflection loss (*R*_*L,min*_) values, attenuation constant (*α*), impedance match degree (Δ), radar cross section (RCS) simulation, and computational analysis were presented in the supporting information.

## Results and Discussion

### Characterization of Co/Co_3_O_4_@HCNs

**Fig. 1:**
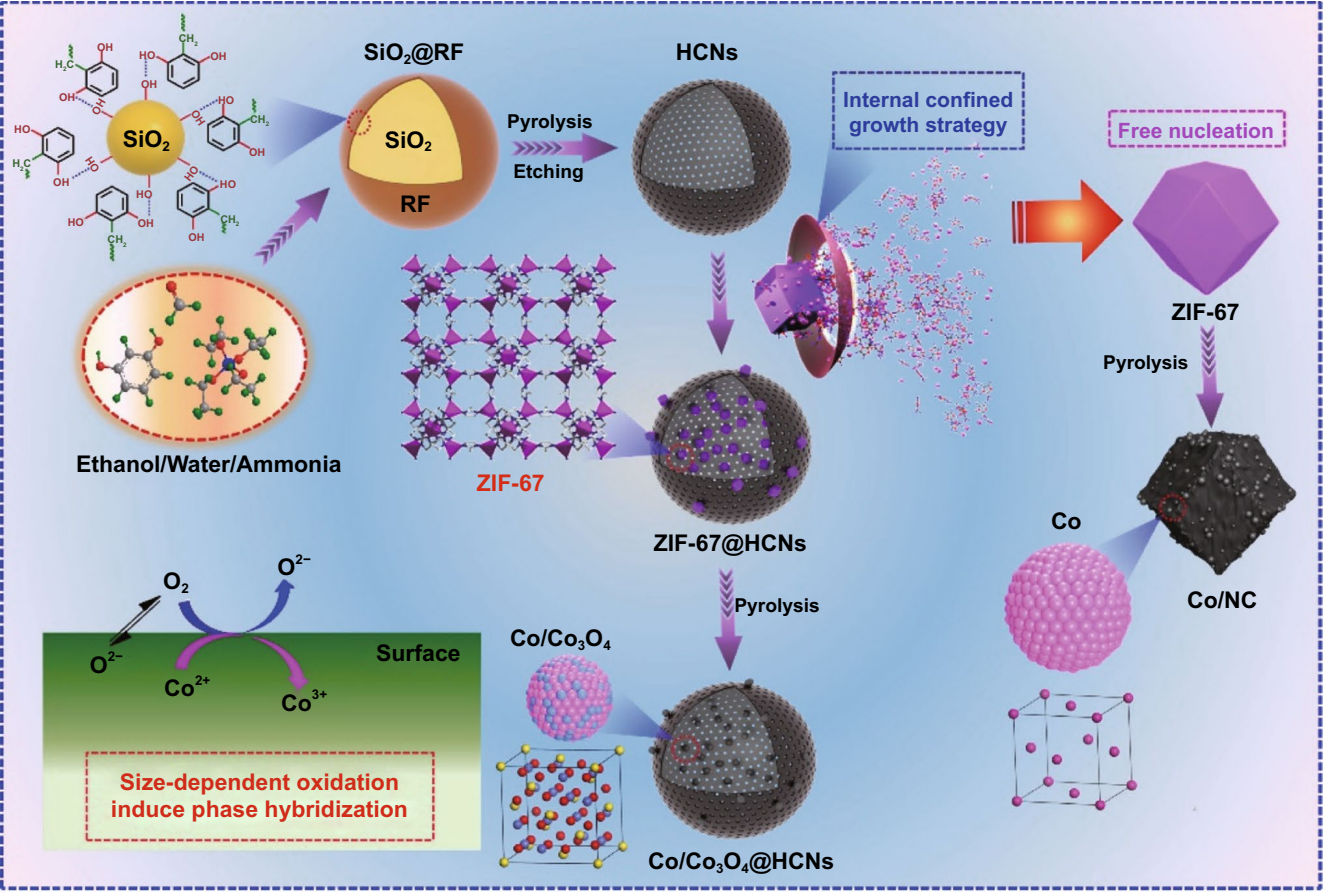
The schematic illustration for the synthetic procedure of Co/Co_3_O_4_@HCNs

**Fig. 2:**
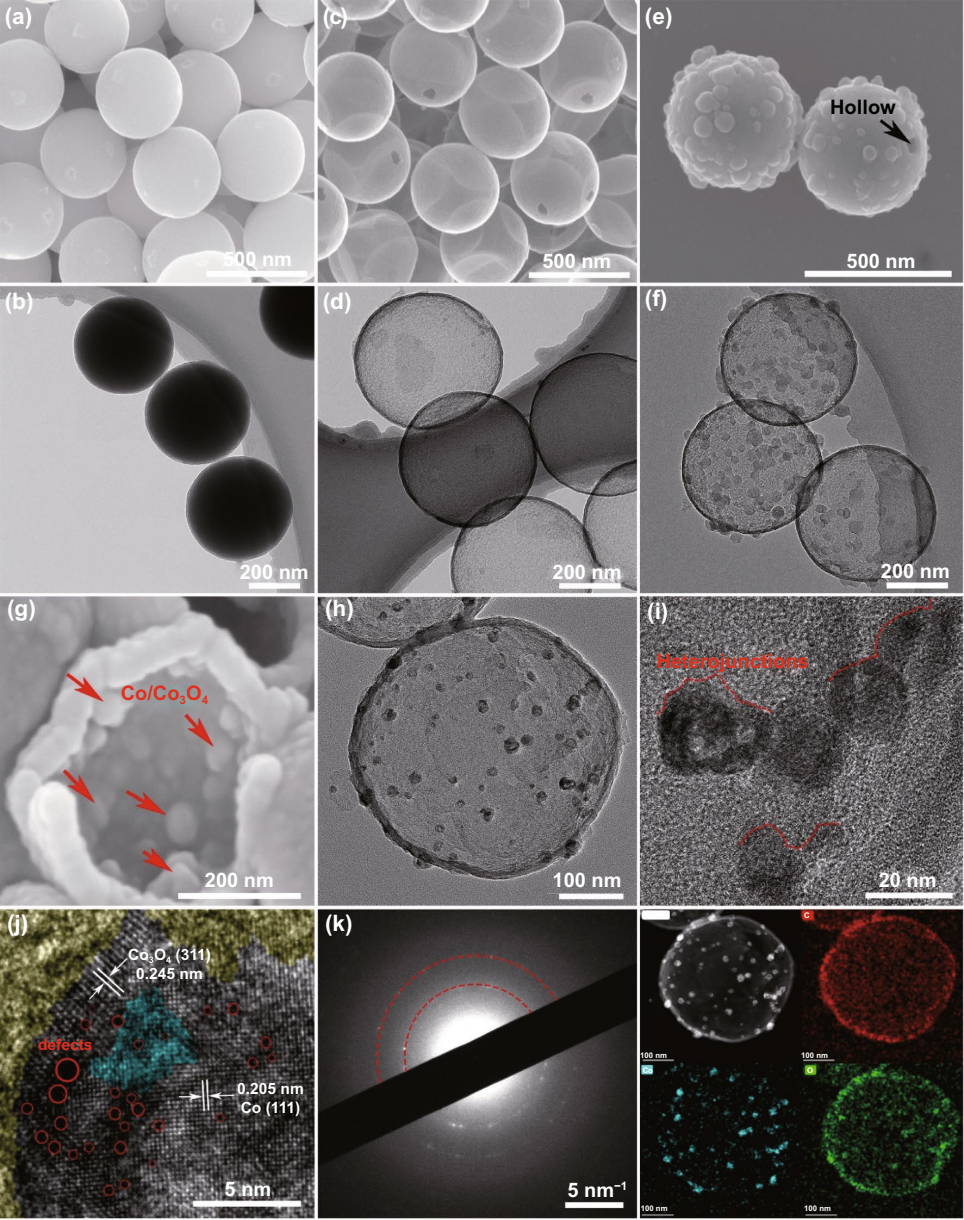
SEM images and TEM images of **a-b** SiO_2_@RF, **c–d** HCNs**, e–f** ZIF-67@HCNs. **g–k** SEM image, TEM images, HRTEM image, SAED pattern, and corresponded elemental mapping images of Co/Co_3_O_4_@HCNs

The overall synthetic process is schematically illustrated in Fig. 1. Firstly, uniform core–shell SiO_2_@resorcinol–formaldehyde (SiO_2_@RF) spheres, with smooth surface and average diameter of ~ 430 nm, are formed by using a modified Stöber method with the coexistence of resorcinol and formaldehyde in an alkaline system, as confirmed by SEM and TEM images in Fig. 2a–b. The synthesized SiO_2_@RF spheres are composed of C, N, O, and Si elements (Fig. S1), and the corresponding element distribution maps imply that these SiO_2_ spheres are completely wrapped by RF matrix. FTIR illustrates the –OH groups on SiO_2_ spheres interact with RF resin via hydrogen bonds in Fig. S2 which further confirms the formation of core–shell SiO_2_@RF spheres. Secondly, HCNs, with an internal hollow cavity and thin carbon shell of about 15 nm, are obtained via transforming the RF matrix into carbon shell under a carbonization process, and the sacrificial SiO_2_ templates are completely removed by etching in hot NaOH solution for 10 h, as shown in Figs. 2c–d and S3. The mesoporous characteristic of the carbon shell, with an optimal pore-size distribution of 6 nm, provides an effective channel for Co^2+^ and 2-MeIm to enter the internal void (Fig. S4), and the large cavity with a diameter of ~ 400 nm can be used as confined cages for the growth of ZIF-67 particles. If etching time is only 2 h, the shadows in Fig. S5 reveal that only a few of the sacrificial SiO_2_ templates are removed. Thirdly, Co^2+^ and 2-MeIm ions can easily infiltrate into the internal space of HCNs owing to the high penetration and porous characteristic of the shell, and both of them assemble to small-size ZIF-67 particles (Figs. 2e–f, S6 and Movie S1). In particular, the presence of negligible N atoms originated from the carbonization of residual ammonia in HCNs can anchor Co^2+^ species by the electrostatic action, so it is clear to observe that some ZIF-67 crystals individually deposit on the outside surface of HCNs [33]. XRD pattern of ZIF-67@HCNs shows the same standard crystal structure to that of ZIF-67 (Fig. S7), suggesting that ZIF-67 crystals are encapsulated into HCNs. In order to demonstrate the spatial confined growth effect, the morphologies of ZIF-67 crystals in a free nucleation environment without adding HCNs are shown in Fig. S8. Under an uninhibited environment, it is freedom for ZIF-67 to grow and larger crystals can be obtained compared with these confined ZIF-67 crystals. Finally, these confined ZIF-67 crystals are in situ transformed into small-size Co/Co_3_O_4_ derivatives via a carbonization process owing to the partial oxidation, resulting in the formation of Co/Co_3_O_4_@HCNs. The overall morphology does not differ significantly from that of HCNs spheres except that reduced Co/Co_3_O_4_ particles, with the diameter in the range of 10–20 nm, are confined in the internal void, as presented in Fig. 2g–h. HRTEM images are carried out to clarify the coherent heterojunctions and phase hybridization by analyzing the crystal lattice. In Fig. 2i, it is clear that the coherent heterojunctions between small-size derivatives and carbon matrix are noted. In detail, as shown in Fig. 2j, the lattice spacing of 0.205 nm corresponds to the (111) plane of Co crystal and the spacing of 0.245 nm is assigned to the (311) plane of Co_3_O_4_, respectively. The clear grain boundaries with phase inversion, as indicated by blue area, imply that Co and Co_3_O_4_ are separated with each other, verifying the phase conversion and generating phase hybridization with obvious coherent interfaces [34]. Simultaneously, a large number of defects can be observed (red circles), and both of the coherent interfaces and defects will trigger strong dipolar/interfacial polarization. In addition, SAED pattern confirms the polycrystals characteristics in Fig. 2k and the elemental mapping images suggest the presence of C, O, and Co elements. For the Co/NC composites derived from ZIF-67 crystals in a free nucleation without adding HCNs (Fig. S9), it is clear that reduced Co nanoparticles, enclosed by graphitic carbon layer with polycrystals, tend to aggregate with obvious crystal region and less defects, resulting in larger size compared with confined Co/Co_3_O_4_ derivatives. Under atmosphere condition without protection, metal particles with smaller size will produce more unavoidable defects and active sites, which are expected to be oxidized easily by providing a channel for oxygen incorporation compared with these ZIF-67 derivatives in a free nucleation environment without adding HCNs, in which the larger reduced Co particles are protected by the graphitic layers [35], leading to phase hybridization (Co/Co_3_O_4_) due to the size-dependent oxidation and adjustable polarization behavior between heterojunctions and coherent interfaces.

**Fig. 3:**
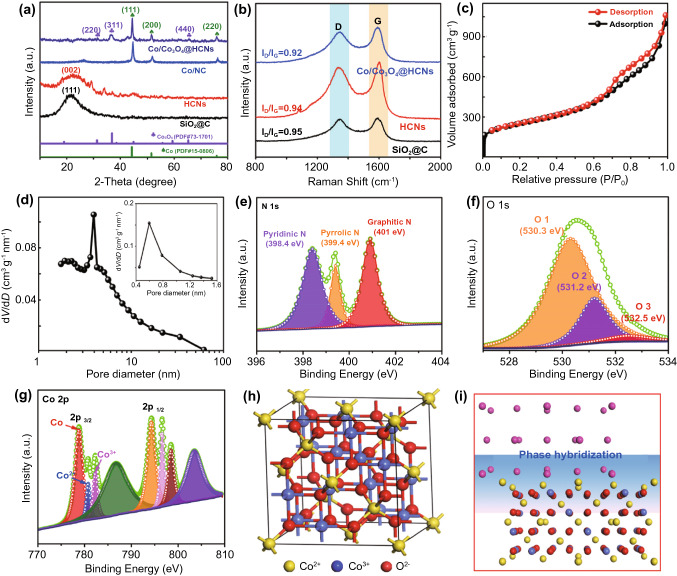
**a** XRD patterns of SiO_2_@C, HCNs, Co/NC, and Co/Co_3_O_4_@HCNs, **b** Raman spectra of SiO_2_@C, HCNs, and Co/Co_3_O_4_@HCNs, **c** N_2_ absorption–desorption isotherm, **d** pore-size distribution, **e** N 1*s* spectrum, **f** O 1*s* spectrum, and **g** Co 2*p* spectrum of Co/Co_3_O_4_@HCNs. **h** The simplified spinel structure of Co_3_O_4_. **i** The multiphase interfacial structure of Co/Co_3_O_4_

The crystal structure is identified by XRD pattern in Fig. 3a. The results prove that the diffraction peak located at ~ 22° for SiO_2_@C demonstrates the amorphous structure and the broad diffraction peak for HCNs is indexed to the (002) plane of carbon matrix. Obviously, the three diffraction peaks of Co/NC at 44.6°, 51.6°, and 75.8° match well with standard cubic Co [*Fm-3 m*] (PDF#15–0806) crystals. For Co/Co_3_O_4_@HCNs, except for the diffraction peaks of cubic Co crystals, the other diffraction peaks are assigned to cubic Co_3_O_4_ [*Fd-3 m*] (PDF#73–1701) because the boundary of cubic Co preferentially promotes the orientation of cubic Co_3_O_4_ with identical crystal symmetry and lower interfacial energy due to the size-dependent oxidation. Interesting, it is noted that the diffraction peak of (111) slightly shifts to low degree with decrease in the particles sizes, which is attributed to the fact that some Co atoms are substituted by O atoms in the lattice, leading to the formation of phase hybridization [36]. Raman spectra are utilized to investigate the presence of Co_3_O_4_ and defects in carbon matrix. As shown in Fig. S10, F_2g_ (1) mode at 191 cm^−1^ is assigned to the translation of the CoO_4_ unit, E_g_ (473 cm^−1^) with other two F_2g_ modes (514 cm^−1^ and 612 cm^−1^) represents the vibrations of tetrahedral and octahedral sites, and A_1g_ mode at 676 cm^−1^ matches well the symmetric stretching of Co^3+^-O and the bending of Co^2+^-O, suggesting the formation of Co_3_O_4_ with spinel lattice [37]. In Fig. 3b, it is clear that the *I*_*D*_/*I*_*G*_ values of SiO_2_@C and HCNs are as high as 0.95 and 0.94 due to the defects in carbon skeleton and incomplete graphitization, while the presence of Co particles promotes the graphitization of carbon, resulting in lower value of 0.92 for Co/Co_3_O_4_@HCNs. N_2_ adsorption–desorption isotherms are conducted to characterize the porous characteristics. As presented in Fig. 3c, the type-I–V curve in the middle region of the pressure suggests the presence of mesopores, and the increased hysteresis loops at low/high pressure indicate the coexistence of micro/macropores. The pore-size distribution (Fig. 3d) implies the significant amount of mesopores with an optimal diameter of ~ 3.9 nm and the coexistence of micropores (0.4–1.2 nm). The specific surface area and pore volume of Co/Co_3_O_4_@HCNs are measured to be 739.3 m^2^ g^−1^ and 1.5491 cm^3^ g^−1^, larger than that of Co/NC (Figs. S11 and S12). XPS survey spectrum in Fig. S13a confirms the presence of C, N, O, and Co elements. C 1*s* spectrum (Fig. S13b) is deconvoluted into four peaks of C=C, C–N, C–O, and O–C–O, respectively. More detailed information of N atoms is depicted in Fig. 3e, in which N 1*s* spectrum corresponds to three peaks, pyridinic N at 397.9 eV, pyrrolic N at 399.3 eV, and graphitic N at 400.9 eV, suggesting the formation of N-doped carbon matrix. In Fig. 3f, the O1 peak at 530.3 eV is indexed to the lattice oxygen in metal oxide (Co–O), the O2 peak at 531.2 eV belongs to the surface oxygen vacancies and the small O3 peak at a higher binding energy of 532.5 eV is attributed to the unavoidable adsorbed/residual water molecules or C-O bond in carbon skeleton [38]. High-resolution Co 2*p* spectrum in Fig. 3g fits well with the corresponding peaks of magnetic Co (778.8 and 794.3 eV) and different oxidation states (Co^2+^ at 780.8 eV and Co^3+^ at 782.3 eV), implying the coexistence of Co and Co_3_O_4_. The simplified spinel structure of Co_3_O_4_ in Fig. 3h indicates that Co^2+^ with tetrahedral coordination and Co^3+^ with octahedral coordination share the unit cell occupancies with O^2−^, the interfacial structure indicates the phase hybridization between Co and Co_3_O_4_ in the coherent interfaces (Fig. 3i), and thus, the multiple phases and abundant heterojunctions with different frequency responses are desired for dielectric polarization, which will be clearly analyzed below.

### Microwave Absorption Performance of Co/Co_3_O_4_@HCNs

**Fig. 4:**
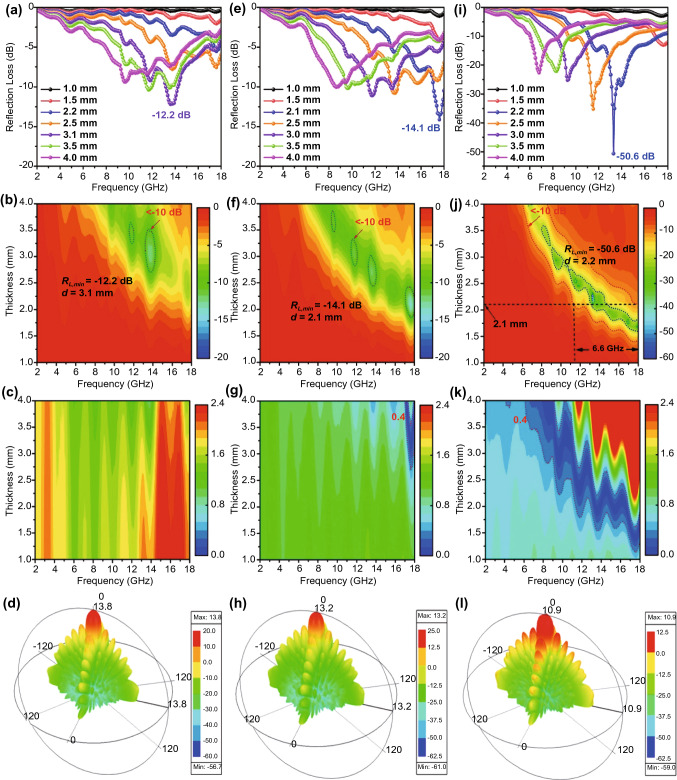
*R*_*L*_ values, 2D projection, Δ maps, and 3D spherical coordinate diagrams of HFSS simulation analysis for **a–d** SiO_2_@C, **e–h** HCNs, and **i-l** Co/Co_3_O_4_@HCNs

The microwave absorption of SiO_2_@C, HCNs, and Co/NC@HCNs with 20 wt% loading content is presented in Fig. 4. The results display that the minimum reflection loss (*R*_*L,min*_) values of SiO_2_@C (Fig. 4a–c) and HCNs (Fig. 4e–g) are merely −12.2 and −14.1 dB due to the impedance mismatch (the larger |Δ| value with large area means unmatched impedance), and thus, most of the incident microwave will reflect at the surface of the absorbers. Besides, the separated closed circles in the *R*_*L*_ contour plots demonstrate the narrow effective absorption bandwidth (EAB). Obviously, with regard to Co/Co_3_O_4_@HCNs as shown in Fig. 4i–k, the synergistic loss mechanism and hollow engineering can indeed favor the impedance characteristic and absorption capacity simultaneously; thus, the area of the |Δ| value close to zero is larger than that of SiO_2_@C and HCNs, the *R*_*L,min*_ value significantly increases to −50.6 dB with a thickness of 2.2 mm, and the EAB regions expand into strips, reaching 6.6 GHz at 2.1 mm. Moreover, the lower RCS simulation value of Co/Co_3_O_4_@HCNs demonstrates that electromagnetic scattering is effectively suppressed after coating absorbers on the metal plate (Fig. 4e, h, l), especially at zero degree, indicating good microwave absorbing ability. From above discussion, we can conclude that optimized absorption capability and preferable bandwidth are simultaneously achieved for Co/Co_3_O_4_@HCNs compared with SiO_2_@C, HCNs, and Co/NC (the *R*_*L,min*_ value of −32.3 dB and EAB of 4.1 GHz, Fig. S14), implying the attenuation performance can be effectively manipulated by the phase hybridization owing to the size-dependent oxidation aforementioned.

**Fig. 5:**
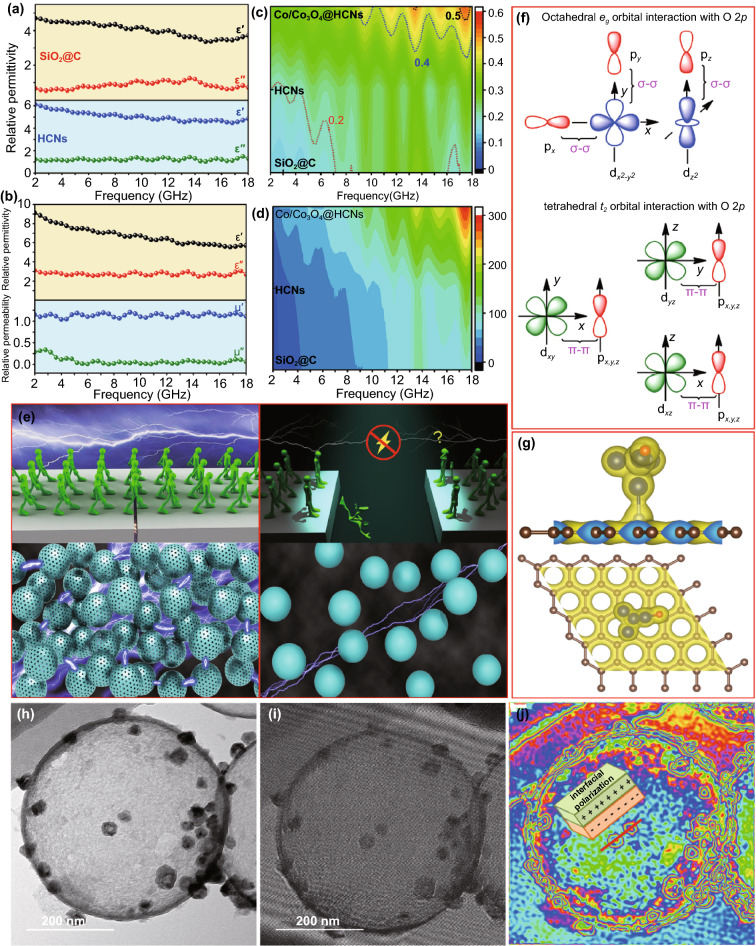
**a-c** The electromagnetic parameters and **d** attenuation constant of SiO_2_@C, HCNs, and Co/Co_3_O_4_@HCNs. **e** Electron transport mechanism in hollow and solid structure**. f** Illustration of tetrahedral and octahedral Co 3*d*–O 2*p* interactions. **g** The simulation results of density map, **h** TEM image, **j** hologram **i** and charge density map of Co/Co_3_O_4_@HCNs

It is well known that the absorption performance is determined by the two electromagnetic parameters in the frequency range of 2–18 GHz. As depicted in Fig. 5a, the complex permittivity real part (*ε*') of SiO_2_@C and HCNs decreases slightly in the whole frequency region, and the lower imaginary part (*ε*") with almost constant values is attributed to the existence of insulated SiO_2_ core and amorphous carbon shell with poor electrical conductivity, resulting in unmatched impedance and unsatisfied dielectric loss. The complex permeability (*μ′*≈1 and *μ″*≈0) indicates a negligible magnetic loss. On the contrary, small-size Co/Co_3_O_4_ derivatives with defects, phase hybridization, and large numbers of coherent interfaces will induce dipolar/interfacial polarization, and thus, it is obvious that Co/Co_3_O_4_@HCNs show the obvious enhanced complex permittivity (*ε*' and *ε*") [39]. In order to clarify the underlying relationship between phase hybridization and dielectric behaviors in detail, conduction loss (*ε*_*c*_") and polarization loss (*ε*_*p*_") of Co/Co_3_O_4_@HCNs, separated from *ε*", are investigated. In general, dielectric loss is classified into conduction loss (ε_c_") and polarization loss (ε_p_") according to Debye theory, and the imaginary part *ε*" is described as follows:

1 $$ \varepsilon^{{{\prime \prime }}} { = }\varepsilon_{{\text{p}}}^{{{\prime \prime }}} + \varepsilon_{c}^{{{\prime \prime }}} = \frac{{\varepsilon_{s} - \varepsilon_{\infty } }}{{1 + \omega^{2} \tau^{2} }}\omega \tau + \frac{\sigma }{{\omega \varepsilon_{0} }} $$

2 $$ \varepsilon_{c}^{{{\prime \prime }}} = \frac{\sigma }{{\omega \varepsilon_{0} }} $$

where *σ* is the electrical conductivity, *ω* is the angular, and *ε*_*0*_ is the permittivity in vacuum. As shown in Fig. S15, it is obvious that the *ε*_*c*_" values exhibit a downward trend with increasing the frequency and the *ε*_*p*_" values are obviously higher than that of *ε*_*c*_" in the whole frequency range of 2–18 GHz, implying polarization loss (dipolar and interfacial polarization) is shown to be dominant in determining dielectric behavior. Therefore, encapsulating small-size MOFs derivatives into HCNs via spatial confinement is an effective strategy to realize high dielectric loss (Fig. 5c). In the *μ″* curve, the enhanced anisotropy energy induced by small-size Co/Co_3_O_4_ particles shifts the frequency to gigahertz; thus, the resonance peaks at low frequency originate from the natural ferromagnetic resonance, and the resonance peaks at high frequency belong to exchange resonance because the crystal size of derivatives is close to the exchange length [40]. Except for promoted impedance matching, it is obvious that the attenuation constant of Co/Co_3_O_4_@HCNs is larger than SiO_2_@C and HCNs over the whole investigated frequency in Fig. 5d, further implying stronger microwave attenuation ability [41]. For practical applications, it is obvious that the specific reflection loss (SRL) of Co/Co_3_O_4_@HCNs, based on the formula of SRL_l_ =|*R*_*L,min*_|/loading content and SRL_lt_ =|*R*_*L,min*_|/(loading content × layer thickness), remarkably surpasses most reported MOFs-derived counterparts (Fig. S16) due to strong *R*_*L,min*_, low loading content as well as thin layer thickness, implying the promising candidates for lightweight microwave absorbers.

### Analysis of Microwave Absorption Mechanism

In general, the detailed dissipation microwave mechanism of Co/Co_3_O_4_@HCNs is closely related to matched impedance, enhanced dielectric polarization, improved magnetic loss as well as the synergistic loss capacity, which can be systematically clarified as follows [42–44]. First, it is reasonable to speculate that these Co/Co_3_O_4_ derivatives embedded inside carbon matrix favor dielectric–magnetic balance, and hollow engineering drastically decreases the interfacial impedance gap at the interface of absorbers–air [45], and both of them result in optimized impedance characteristic and less backscattered microwave, which is the critical precondition for subsequent microwave attenuation. Second, it is nonnegligible that carbon shell with hollow structure can build more efficient electronic transmission channel by the physical contacts compared with solid counterparts in the same mass condition, as illustrated in Fig. 5e, which facilitates the electronic transmission and consumes the microwave energy [46], resulting in dominant role in conduction loss because the loss that stems from Co/Co_3_O_4_ derivatives can be ignored owing to the small size. Besides, the intricate conductive network spontaneously responds to the incident microwave and intensely induces time-varying electromagnetic field-induced current in the circular resistive network, converting electromagnetic energy into thermal energy [47, 48]. Third, polarization loss primarily originated from dipolar/interfacial polarization becomes the dominant factor in determining the dielectric behavior. By examining the effect of dipolar polarization on polarization loss, the so-called ionocovalent bonding nature of Co_3_O_4_ is shown in Fig. 5f, where the spatial overlap from the Co 3*d* orbital to O 2*p* orbital, especially Co^3+^, strongly interacts with O 2*p*, strengthens the bond, and leads to electron polarization. The coexistence of ion hybridization in Co_3_O_4_, induced defects, heteroatoms, and residual functional groups, as proved by the XPS analysis and simulated calculations (Fig. 5g), will displace the local electron density and trigger dielectric dipole oscillations [49], resulting in enhanced dipolar polarization. Besides, because of the compatibility between Co and Co_3_O_4_ with same cubic phases, the transformation promotes the phase hybridization with coherent interfaces [50, 51]. Combining with heterojunctions and hierarchical pores, strong interfacial polarization can be achieved, which are expressed by the typical Lorentz off-axis electron holograms in Fig. 5h–j. It is clear that the electrical characteristics, among the coherent interfaces, heterojunctions as well as hierarchical pores, vary significantly and display different charge densities because of the redistribution, transfer, or accumulation, which act as the topological conditions to produce capacitor-like structures at the heterogeneous interfaces and generate strong interfacial loss [52, 53]. The multiple Debye relaxation polarization also can be verified by the Cole–Cole plots with different resonance peaks in Fig. S17 [54], but it is difficult to precisely identify the exact dipolar or interfacial polarization at present. Profiting from the feature of strong polarization behavior, the Co/Co_3_O_4_@HCNs absorbers will exhibit satisfied dielectric loss. Fourth, with respect to magnetic loss, it is no doubt that it is mainly dominated by ferromagnetic resonance and eddy current loss in 2–18 GHz. Figure S18 shows the hysteresis loops of Co/NC and Co/Co_3_O_4_@HCNs. It reveals that the saturation magnetization (*M*_*s*_) and coercivity (*H*_*c*_) values are measured to be 43.6 emu g^−1^, 28.1 emu g^−1^ and 309.4 Oe, 281.1 Oe, respectively. The moderate lower *M*_*s*_ value of Co/Co_3_O_4_@HCNs can be explained by the additional nonmagnetic hollow cavity, and the decreased *H*_*c*_ value is attributed to the smaller grain size than the critical value (~ 70 nm) for Co particles under the identical crystalline structure and approximate shape anisotropy, which is favorable for the initial permeability and magnetic loss [55]. The magnetic loss induced by eddy current effect is characterized by the coefficient value (*μ*″(*μ*′) ^−2^*f*^−1^) as depicted in Fig. S19, and the value is almost constant when the frequency is over 7 GHz, implying that eddy effect also plays a fundamental role in magnetic loss at this frequency region. In addition, the integrated synergistic effect can effectively strengthen multiple loss coordination by making full use of dielectric loss and magnetic loss, and the hierarchical pores trigger multiple scattering and propagated pathway; both of them are beneficial to absorption capability [56].

## Conclusions

In summary, we have proposed a spatial confined growth strategy to encapsulate small-size ZIF-67 crystals into HCNs, in which multiple derivatives are confined in hollow cavity of HCNs by the spatial confinement effect. Being determined by size-dependent oxidation, dielectric polarization originated from phase hybridization, abundant heterojunctions, and hierarchical pores is dominant in dielectric behavior. Moreover, the internal hollow cavity of HCNs adjusts impedance characteristics and multiple scattering contributes absorption attenuation to some degree. Particularly, an optimal absorption ability of −50.6 dB and effective bandwidth of 6.6 GHz are achieved. The spatial confinement strategy demonstrates a new strategy for manipulating the size of MOFs derivatives, and the results provide a theoretical guideline for optimizing polarization behaviors by the size-dependent phase hybridization.

## Supplementary Information

Below is the link to the electronic supplementary material.

Supplementary file1 (MP4 570 kb)

Supplementary file2 (PDF 1738 kb)

## References

[1] Lv H, Yang Z, Wang P, Ji G, Song J (2018). A voltage-boosting strategy enabling a low-frequency, flexible electromagnetic wave absorption device. Adv. Mater..

[2] Wu Z, Cheng HW, Jin C, Yang B, Xu C (2021). Dimensional design and core-shell engineering of nanomaterials for electromagnetic wave absorption. Adv. Mater..

[3] Che R, Peng L, Duan X, Chen Q, Liang X (2004). Microwave absorption enhancement and complex permittivity and permeability of Fe encapsulated within carbon nanotubes. Adv. Mater..

[4] Sun H, Che R, You X, Jiang Y, Yang Z (2014). Cross-stacking aligned carbon-nanotube films to tune microwave absorption frequencies and increase absorption intensities. Adv. Mater..

[5] Wen B, Cao M, Lu M, Cao W, Shi H (2014). Reduced graphene oxides: light-weight and high-efficiency electromagnetic interference shielding at elevated temperatures. Adv. Mater..

[6] Balci O, Polat EO, Kakenov N, Kocabas C (2015). Graphene-enabled electrically switchable radar-absorbing surfaces. Nat. Commun..

[7] Iqbal A, Shahzad F, Hantanasirisakul K, Kim MK, Kwon J (2020). Anomalous absorption of electromagnetic waves by 2D transition metal carbonitride Ti_3_CNT_x_ (MXene). Science.

[8] Bi YX, Ma ML, Liu YY, Tong ZY, Wang RZ (2021). Microwave absorption enhancement of 2-dimensional CoZn/C@MoS_2_@PPy composites derived from metal organic framework. J. Coll. Interface Sci..

[9] Ning M, Jiang P, Ding W, Zhu X, Tan G (2021). Phase manipulating toward molybdenum disulfide for optimizing electromagnetic wave absorbing in gigahertz. Adv. Funct. Mater..

[10] Liu J, Che R, Chen H, Zhang F, Xia F (2012). Microwave absorption enhancement of multifunctional composite microspheres with spinel Fe_3_O_4_ cores and anatase TiO_2_ shells. Small.

[11] Du Y, Liu W, Qiang R, Wang Y, Han X (2014). Shell thickness-dependent microwave absorption of core-shell Fe_3_O_4_@C composites. ACS Appl. Mater. Interfaces.

[12] Liu Q, Cao Q, Bi H, Liang C, Yuan K (2016). CoNi@SiO_2_@TiO_2_ and CoNi@Air@TiO_2_ microspheres with strong wideband microwave absorption. Adv. Mater..

[13] Zhang X, Zhu J, Yin P, Guo A, Huang A (2018). unable high-performance microwave absorption of Co_1-x_S hollow spheres constructed by nanosheets within ultralow filler loading. Adv. Funct. Mater..

[14] Li X, Yin X, Song C, Han M, Xu H (2018). Self-assembly core-shell graphene-bridged hollow MXenes spheres 3D foam with ultrahigh specific EM absorption performance. Adv. Funct. Mater..

[15] Zhang Y, Huang Y, Zhang T, Chang H, Xiao P (2015). Broadband and tunable high-performance microwave absorption of an ultralight and highly compressible graphene foam. Adv. Mater..

[16] Huang Z, Chen H, Huang Y, Ge Z, Zhou Y (2018). Ultra-broadband wide-angle terahertz absorption properties of 3D graphene foam. Adv. Funct. Mater..

[17] Liu P, Zhang Y, Yan J, Huang Y, Xia L (2019). Synthesis of lightweight N-doped graphene foams with open reticular structure for high-efficiency electromagnetic wave absorption. Chem. Eng. J..

[18] Wang HY, Sun XB, Yang SH, Zhao PY, Zhang XJ (2021). 3D ultralight hollow NiCo compound@MXene composites for tunable and high-efficient microwave absorption. Nano-Micro Lett..

[19] Qiang R, Du Y, Zhao H, Wang Y, Tian C (2015). Metal organic framework-derived Fe/C nanocubes toward efficient microwave absorption. J. Mater. Chem. A.

[20] Liu W, Shao Q, Ji G, Liang X, Cheng Y (2017). Metal–organic-frameworks derived porous carbon-wrapped Ni composites with optimized impedance matching as excellent lightweight electromagnetic wave absorber. Chem. Eng. J..

[21] Xiang Z, Song Y, Xiong J, Pan Z, Wang X (2019). Enhanced electromagnetic wave absorption of nanoporous Fe_3_O_4_@carbon composites derived from metal-organic frameworks. Carbon.

[22] Liu D, Qiang R, Du Y, Wang Y, Tian C (2018). Prussian blue analogues derived magnetic FeCo alloy/carbon composites with tunable chemical composition and enhanced microwave absorption. J. Coll. Interface Sci..

[23] Feng W, Wang Y, Chen J, Li B, Guo L (2018). Metal organic framework-derived CoZn alloy/N-doped porous carbon nanocomposites: tunable surface area and electromagnetic wave absorption properties. J. Mater. Chem. C.

[24] Huang M, Wang L, Pei K, You W, Yu X (2020). Multidimension-controllable synthesis of MOF-derived Co@N-doped carbon composite with magnetic-dielectric synergy toward strong microwave absorption. Small.

[25] Deng B, Xiang Z, Xiong J, Liu Z, Yu L (2020). Sandwich-like Fe&TiO_2_@C nanocomposites derived from MXene/Fe-MOFs hybrids for electromagnetic absorption. Nano-Micro Lett..

[26] Wang L, Yu X, Li X, Zhang J, Wang M (2020). MOF-derived yolk-shell Ni@C@ZnO Schottky contact structure for enhanced microwave absorption. Chem. Eng. J..

[27] Lv Y, Wang Y, Li H, Lin Y, Jiang Z (2015). MOF-derived porous Co/C nanocomposites with excellent electromagnetic wave absorption properties. ACS Appl. Mater. Interfaces.

[28] Wang S, Xu Y, Fu R, Zhu H, Jiao Q (2019). Rational construction of hierarchically porous Fe-Co/N-doped carbon/rGO composites for broadband microwave absorption. Nano-Micro Lett..

[29] Cheng RR, Wang Y, Di XC, Lu Z, Wang P (2022). Construction of MOF-derived plum-like NiCo@Ccomposite with enhanced multi-polarization for high-efficiency microwave absorption. J. Coll. Interface Sci..

[30] Liu P, Gao S, Wang Y, Huang Y, He W (2020). Carbon nanocages with N-doped carbon inner shell and Co/N-doped carbon outer shell as electromagnetic wave absorption materials. Chem. Eng. J..

[31] Li Z, Han X, Ma Y, Liu D, Wang Y (2018). MOFs-derived hollow Co/C microspheres with enhanced microwave absorption performance. ACS Sustain. Chem. Eng..

[32] Liu P, Gao S, Zhang G, Huang Y, You W (2021). Hollow engineering to Co@N-doped carbon nanocages via synergistic protecting-etching strategy for ultrahigh microwave absorption. Adv. Funct. Mater..

[33] Xiong W, Li H, You H, Cao M, Cao R (2020). Encapsulating metal organic framework into hollow mesoporous carbon sphere as efficient oxygen bifunctional electrocatalyst. Natl. Sci. Rev..

[34] Gao Z, Lan D, Zhang L, Wu H (2021). Simultaneous manipulation of interfacial and defects polarization toward Zn/Co phase and ion hybrids for electromagnetic wave absorption. Adv. Funct. Mater..

[35] Liu Y, Yang F, Zhang Y, Xiao J, Yu L (2017). Enhanced oxidation resistance of active nanostructures via dynamic size effect. Nat. Commun..

[36] Zhang Q, Wang C, Zhang H, Zhang S, Liu Z (2020). Designing ultrahard nanostructured diamond through internal defects and interface engineering at different length scales. Carbon.

[37] Natarajan K, Munirathinam E, Yang TCK (2021). Operando investigation of structural and chemical origin of Co_3_O_4_ stability in acid under oxygen evolution reaction. ACS Appl. Mater. Interfaces.

[38] Xiao Z, Huang Y, Dong C, Xie C, Liu Z (2020). Operando identification of the dynamic behavior of oxygen vacancy-rich Co_3_O_4_ for oxygen evolution reaction. J. Am. Chem. Soc..

[39] Zhang D, Xiong Y, Cheng J, Chai J, Liu T (2020). Synergetic dielectric loss and magnetic loss towards superior microwave absorption through hybridization of few-layer WS_2_ nanosheets with NiO nanoparticles. Sci. Bull..

[40] Li Y, Liu X, Nie X, Yang W, Wang Y (2019). Multifunctional organic–inorganic hybrid aerogel for self-cleaning, heat-insulating, and highly efficient microwave absorbing material. Adv. Funct. Mater..

[41] Quan B, Shi W, Ong SJH, Lu X, Wang PL (2019). Defect engineering in two common types of dielectric materials for electromagnetic absorption applications. Adv. Funct. Mater..

[42] Wang JJ, Yu SL, Wu QQ, Li Y, Li FY (2022). Heterogeneous junctions of magnetic Ni core@binary dielectric shells toward high-efficiency microwave attenuation. J. Mater. Sci. Technol..

[43] Di XC, Wang Y, Lu Z, Cheng RR, Yang LQ (2022). Heterostructure design of Ni/C/porous carbon nanosheet composite for enhancing the electromagnetic wave absorption. Carbon.

[44] Wang Y, Di XC, Lu Z, Cheng RR, Wu XM (2022). Controllable heterogeneous interfaces of cobalt/carbon nanosheets/rGO composite derived from metal-organic frameworks for high-efficiency microwave attenuation. Carbon.

[45] Wang YL, Yang SH, Wang HY, Wang GS, Sun XB (2020). Hollow porous CoNi/C composite nanomaterials derived from MOFs for efficient and lightweight electromagnetic wave absorber. Carbon.

[46] Liu X, Li Y, Sun X, Tang W, Deng G (2021). Off/on switchable smart electromagnetic interference shielding aerogel. Matter.

[47] Zhang D, Wang H, Cheng J, Han C, Yang X (2020). Conductive WS_2_-NS/CNTs hybrids based 3D ultra-thin mesh electromagnetic wave absorbers with excellent absorption performance. Appl. Surf. Sci..

[48] Zhang D, Liu T, Cheng J, Chai J, Yang X (2019). Light-weight and low-cost electromagnetic wave absorbers with high performances based on biomass-derived reduced graphene oxides. Nanotechnology.

[49] Gao S, Wang GS, Guo L, Yu SH (2020). Tunable and ultraefficient microwave absorption properties of trace N-doped two-dimensional carbon-based nanocomposites loaded with multi-rare earth oxides. Small.

[50] Zhang H, Cheng J, Wang H, Huang Z, Zheng Q (2022). Initiating VB-group laminated NbS2 electromagnetic wave absorber toward superior absorption bandwidth as large as 6.48 GHz through phase engineering modulation. Adv. Funct. Mater..

[51] Huang Z, Cheng J, Zhang H, Xiong Y, Zhou Z (2022). High-performance microwave absorption enabled by Co_3_O_4_ modified VB-group laminated VS_2_ with frequency modulation from S-band to Ku-band. J. Mater. Sci. Technol..

[52] Wu Z, Pei K, Xing L, Yu X, You W (2019). Enhanced microwave absorption performance from magnetic coupling of magnetic nanoparticles suspended within hierarchically tubular composite. Adv. Funct. Mater..

[53] Wang J, Liu L, Jiao S, Ma K, Lv J (2020). Hierarchical carbon fiber@MXene@MoS_2_ core-sheath synergistic microstructure for tunable and efficient microwave absorption. Adv. Funct. Mater..

[54] Shu J, Cao M, Zhang M, Wang X, Cao W (2020). Molecular patching engineering to drive energy conversion as efficient and environment-friendly cell toward wireless power transmission. Adv. Funct. Mater..

[55] Che RC, Zhi CY, Liang CY, Zhou XG (2006). Fabrication and microwave absorption of carbon nanotubes/CoFe_2_O_4_ spinel nanocomposite. Appl. Phys. Lett..

[56] Zhang D, Xiong Y, Cheng J, Raza H, Hou C (2021). Construction of low-frequency and high-efficiency electromagnetic wave absorber enabled by texturing rod-like TiO_2_ on few-layer of WS_2_ nanosheets. Appl. Surf. Sci..

