# Supplementary material for: Size-Dependent Oxidation-Induced Phase Engineering for MOFs Derivatives Via Spatial Confinement Strategy Toward Enhanced Microwave Absorption
Source: Nanomicro Lett. 2022 Apr 12;14:102. doi: 10.1007/s40820-022-00841-5 (PMC9005575; doi:10.1007/s40820-022-00841-5)
Supplement: Supplementary file 2 — Supplementary file2 (PDF 1738 kb) [file 40820_2022_841_MOESM2_ESM.pdf]

Supporting Information for

Size-Dependent Oxidation Induced Phase Engineering for MOFs Derivatives via Spatial Confinement Strategy Toward Enhanced Microwave Absorption

Hanxiao Xu[1, #], Guozheng Zhang[1, #], Yi Wang[1], Mingqiang Ning[2, *], Bo Ouyang[3, *], Yang Zhao[4], Ying Huang[1], Panbo Liu[1, *]

[1]School of Chemistry and Chemical Engineering, µNorthwestern Polytechnical University, Xi'an 710129, P. R. China

[2]Key Laboratory of Magnetic Materials and Devices, Ningbo Institute of Materials Technology & Engineering, Chinese Academy of Sciences, Ningbo 315201, P. R. China

[3]MIIT Key Laboratory of Semiconductor Microstructure and Quantum Sensing, Nanjing University of Science and Technology, Nanjing 210094, P. R. China

[4]Department of Mechanical and Materials Engineering, University of Western Ontario, London, Ontario, N6A 5B9, Canada

[#]Hanxiao Xu and Guozheng Zhang contributed equally to this work and should be considered as co-first authors.

*Corresponding authors. E-mail: liupanbo@nwpu.edu.cn (Panbo Liu), ningmingqiang@nimte.ac.cn (Mingqiang Ning), ouyangboyi@njust.edu.cn (Bo Ouyang)

S1 Experimental Sections

The minimum reflection loss ($R_{L,min}$) values were calculated based on the transmission line theory with the electromagnetic parameters (complex permittivity and complex permeability) measured by a HP8510C vector network analyzer in the frequency range of 2-18 GHz:

$$R_L\,(\mathrm{dB}) = 20\log\left|\frac{Z_{in}-Z_0}{Z_{in}+Z_0}\right| \tag{S1}$$

$$Z_{\mathrm{in}} = Z_0\sqrt{\mu_r/\varepsilon_r}\tanh\left[j(2\pi fd/c)\sqrt{\varepsilon_r\mu_r}\right] \tag{S2}$$

where ε_r and μ_r are the relative complex permittivity and permeability, d is the layer thickness, c is the speed of light in free space and f is the frequency.

The attenuation constant (α) is calculated as follows:

$$\alpha = \frac{\sqrt{2}\pi f}{c}\sqrt{(\mu''\varepsilon''-\mu'\varepsilon')+\sqrt{(\mu''\varepsilon''-\mu'\varepsilon')^2+(\mu'\varepsilon''+\mu''\varepsilon')^2}} \tag{S3}$$

The impedance match degree (Δ) is calculated by a delta-function method as follows:

$$|\triangle| = |sinh^2(Kfd) - M| \tag{S4}$$

$$K = \frac{4\pi\sqrt{\varepsilon_r{}'\mu_r{}'}\times\sin\left(\frac{\delta_e+\delta_m}{2}\right)}{c\cos\delta_e\cos\delta_m} \tag{S5}$$

$$M = \frac{4\mu' cos\delta_e \varepsilon' \cos\delta_m}{(\mu' cos\delta_e - \varepsilon' \cos\delta_m)^2 + [\tan(\frac{\delta m - \delta_e}{2})]^2 \times (\mu' cos\delta_e + \varepsilon' \cos\delta_m)^2} \quad (S6)$$

where K and M can be calculated by the relative complex permittivity and the complex permeability from EqS. (S5) and (S6). A delta value ($|\Delta|<0.4$) with a large area indicates a balanced impedance matching degree.

Radar cross section (RCS) simulation with an aluminium plate is selected to measure the ability of electromagnetic scattering and HFSS software is used to perform RCS simulation calculation. Concretely, the obtained SiO_2@C, HCNs and Co/Co_3O_4@HCNs absorbers dispersed in paraffin matrix with 20 wt% are coated on an aluminum plate ($20\times20\times0.5$ cm^3) with a coating layer of 0.18 cm. The polar plots range from -90° to +90° and the simulation frequency is chosen at 10 GHz.

Computational analysis: The DFT calculation was performed to investigate charge density distribution of different models based on Vienna Ab-initio Software Package (VASP) program with Perdew-Burke-Emzerhof (PBE) exchange-correlation functional. Based on our experimental results, we applied graphene model with different lattice spacing and N-doped graphene surface with CoO decoration. The slab is controlled to 25 Å along the c-direction to prevent the interaction of neighboring atoms. The energy cutoff was set to 500 eV with energy and force consistency of 10^{-5} eV and 0.01 eV $Å^{-1}$. The Van der Waals interaction from dynamical correlation was not considered in our system.

S2 Supplementary Results and Discussion

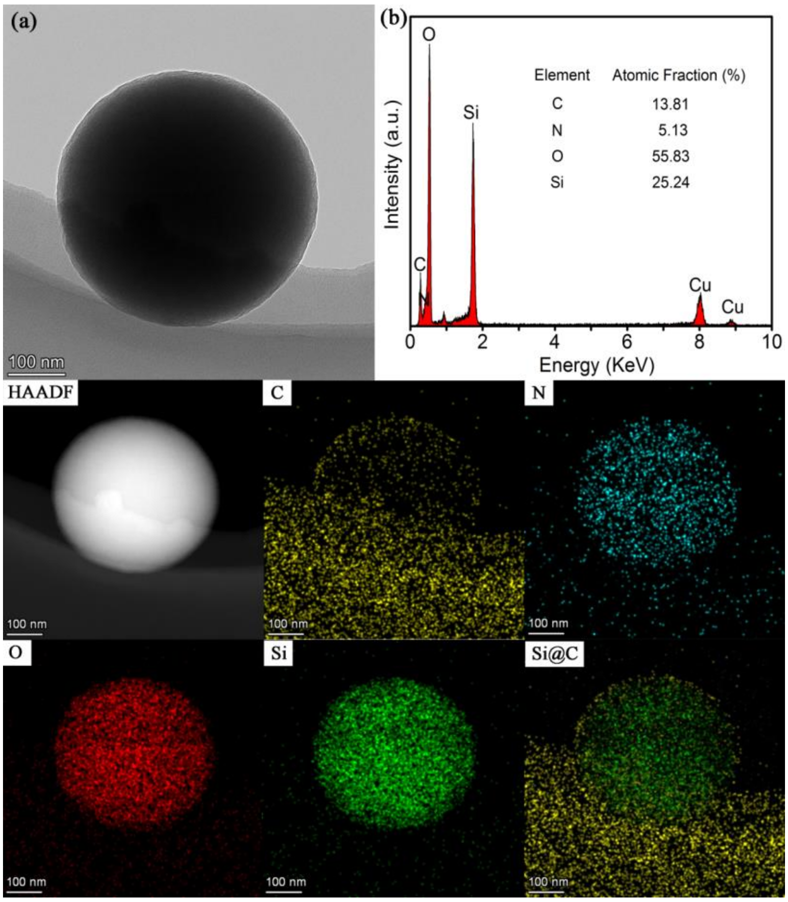

Fig. S1 TEM image **a**, EDS pattern **b** and the corresponding elemental mapping images of core-shell SiO_2@RF spheres

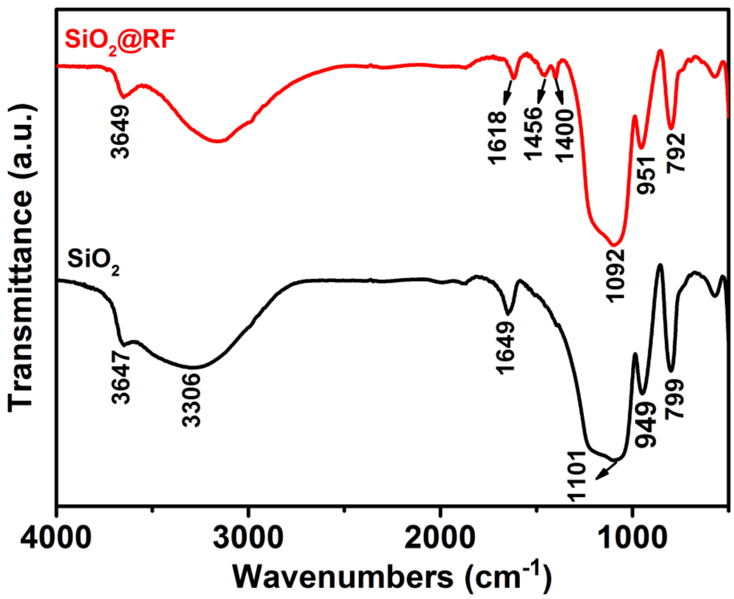

Fig. S2 FTIR spectrum of SiO_2 and SiO_2@RF spheres. For SiO_2, the band at 1101 cm^{-1} is ascribed to the stretching of Si-OH, the band at 949 cm^{-1} is attributed to the stretching of Si-O-Si and the band at 799 cm^{-1} is ascribed to the stretching of Si-O. Because the presence of hydroxy groups (-OH), which can interact strongly with resin through the formation of H bonds in SiO_2@RF spheres

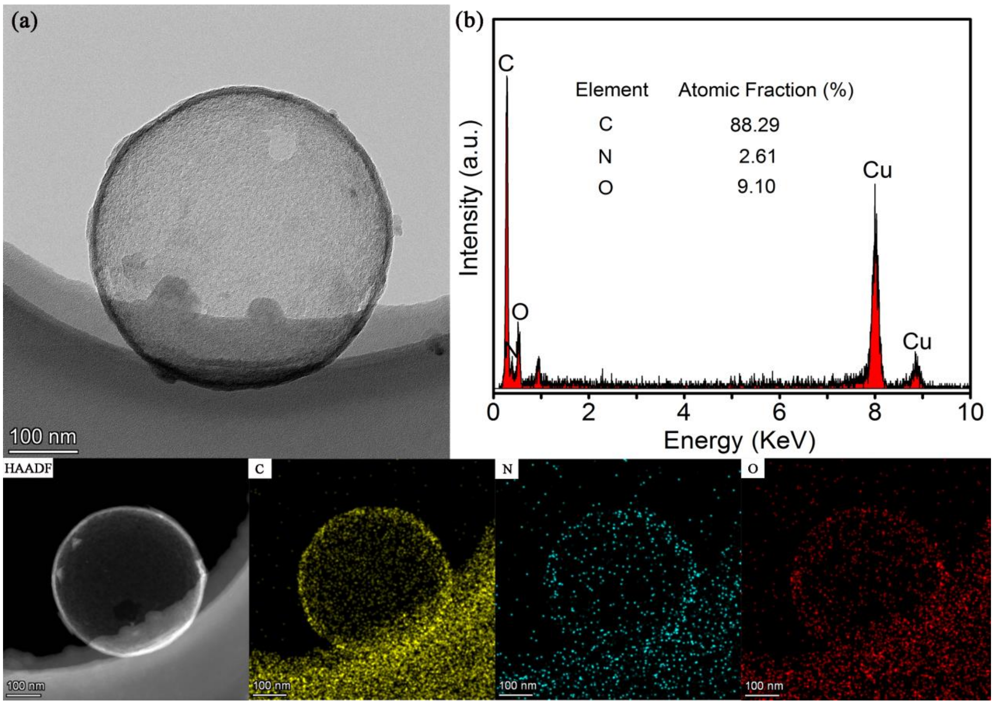

Fig. S3 TEM image **a**, EDS pattern **b** and the corresponding elemental mapping images of HCNs

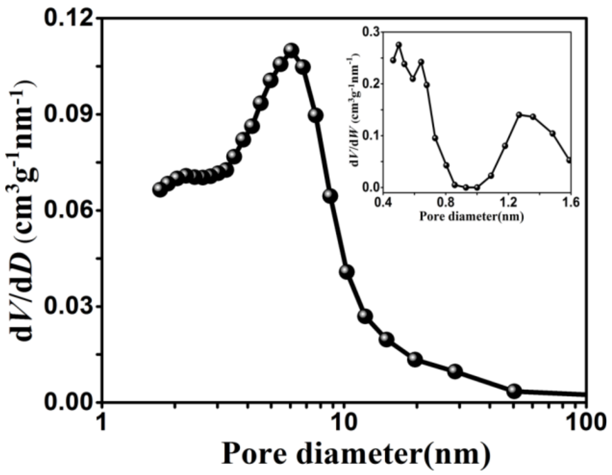

Fig. S4 Pore size distribution of HCNs

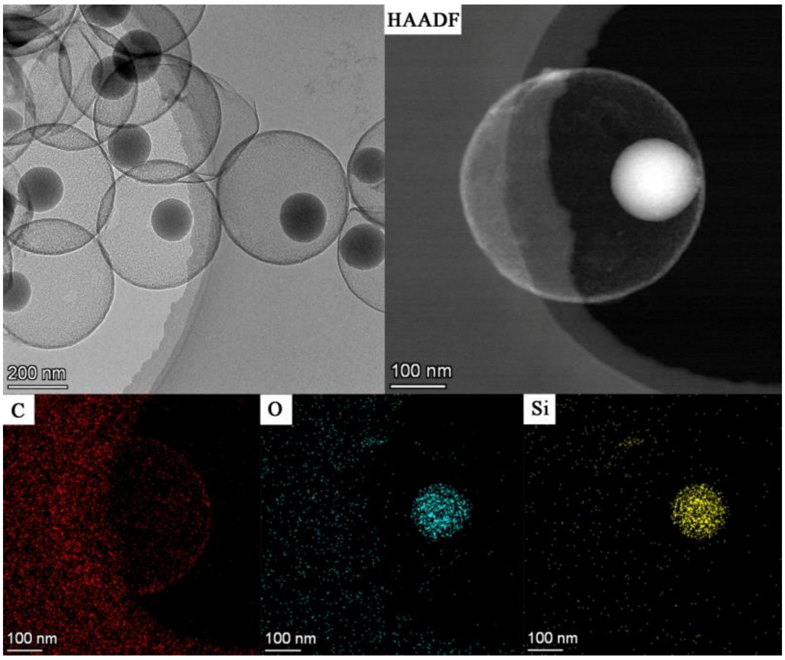

Fig. S5 TEM image of SiO_2@C spheres washing by NaOH solution for 2 h. The corresponding Si and O elemental mapping images indicate that the shadows in the internal cavity are un-removed SiO_2 spheres

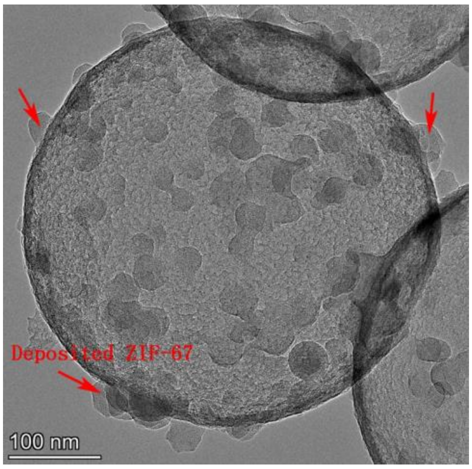

Fig. S6 TEM image of ZIF-67@HCNs

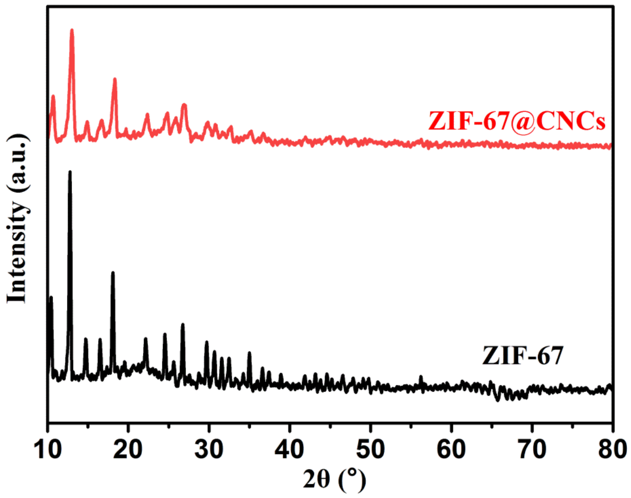

Fig. S7 XRD patterns of ZIF-67 and ZIF-67@HCNs

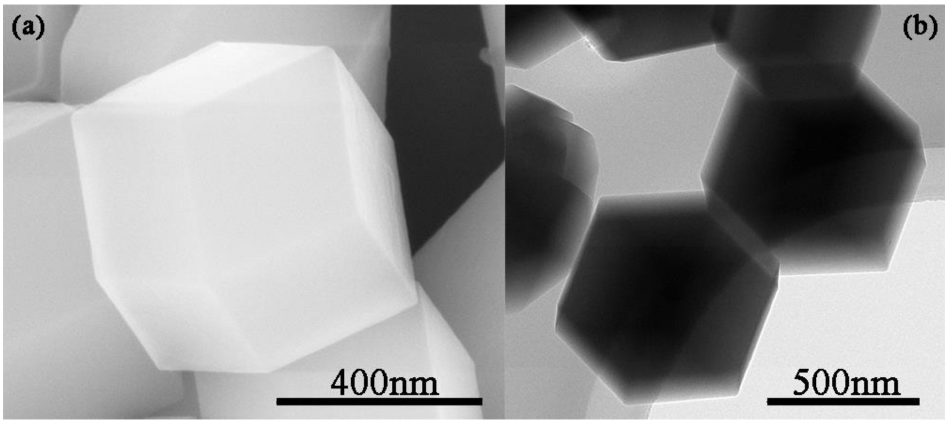

Fig. S8 SEM image **a** and TEM image **b** of ZIF-67 in a free nucleation without adding HCNs

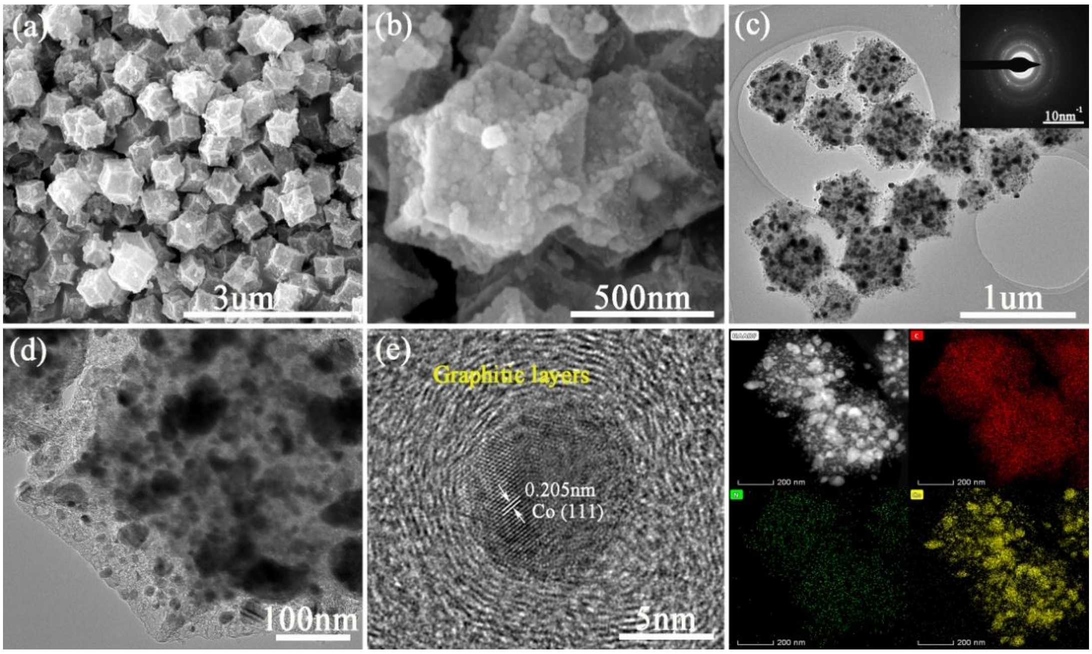

Fig. S9 SEM images **a-b**, TEM images **c-d**, HRTEM image **e** and the corresponding elemental mapping images of the Co/NC composites derived from ZIF-67crystals in a free nucleation without adding HCNs

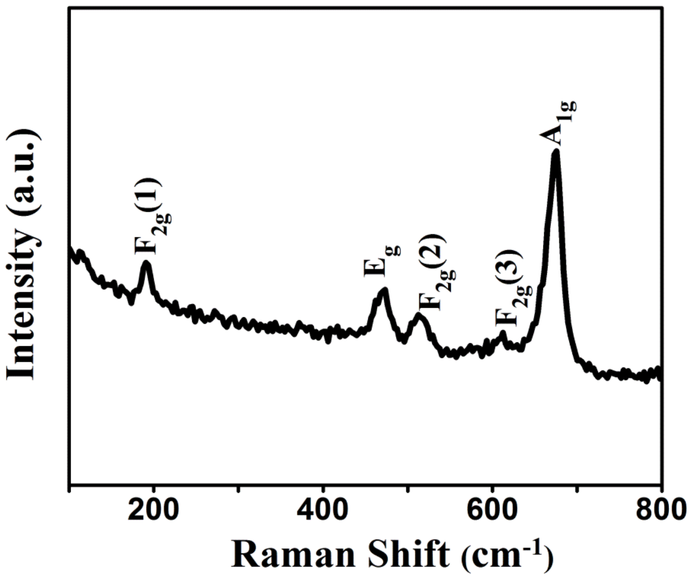

Fig. S10 Raman spectra of Co/Co_3O_4@HCNs

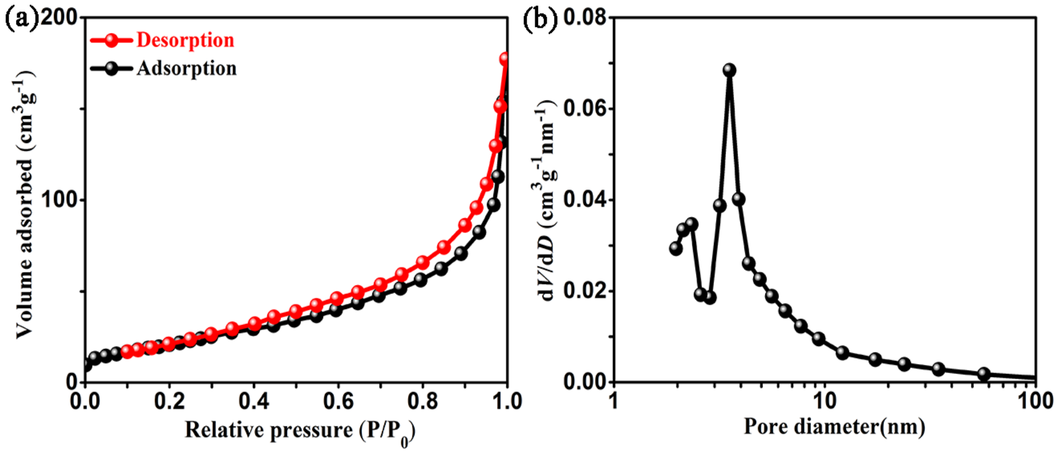

Fig. S11 N_2 absorption-desorption isotherms **a** and pore size distribution **b** of Co/NC

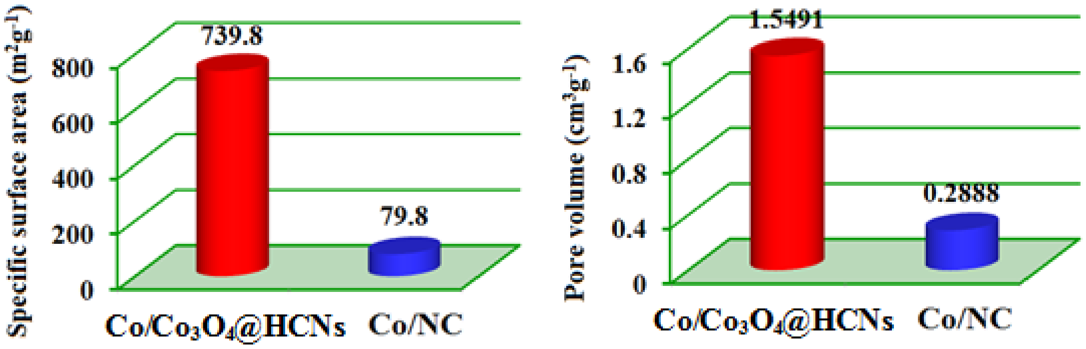

Fig. S12 The specific surface area **a** and pore volume **b** of Co/Co_3O_4@HCNs and Co/NC

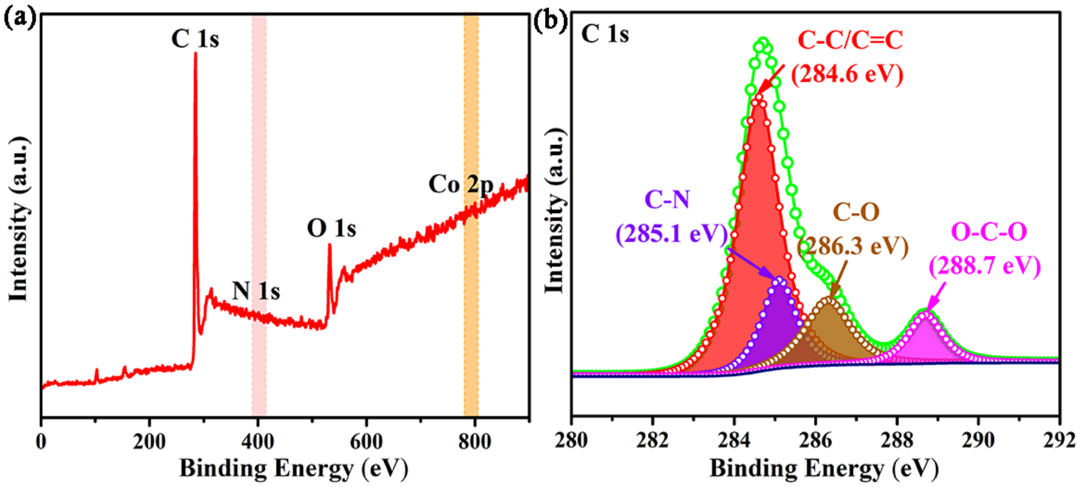

Fig. S13 XPS survey spectrum **a** and C 1s spectrum **b** of Co/Co_3O_4@HCNs

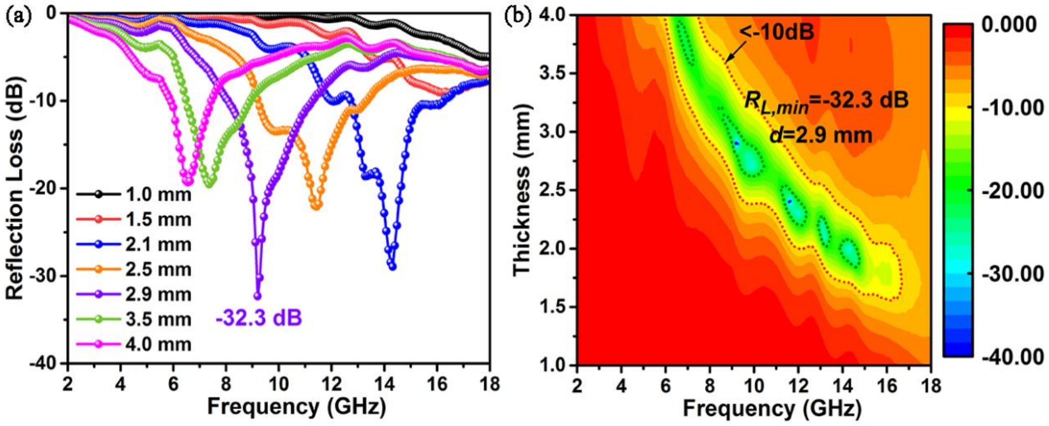

Fig. S14 R_L values and 2D projection of Co/NC

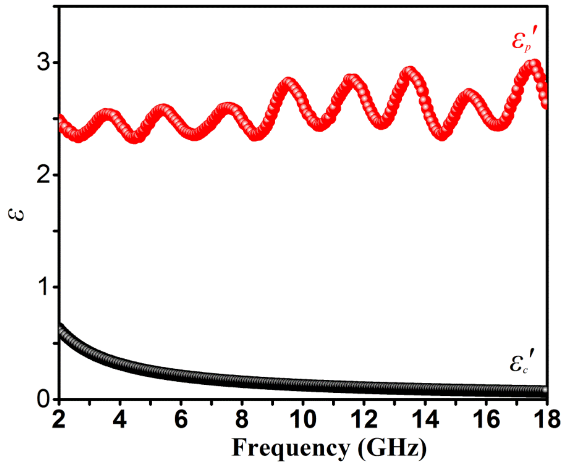

Fig. S15 Frequency dependency of ε_c'' and ε_p'' for Co/Co_3O_4@HCNs

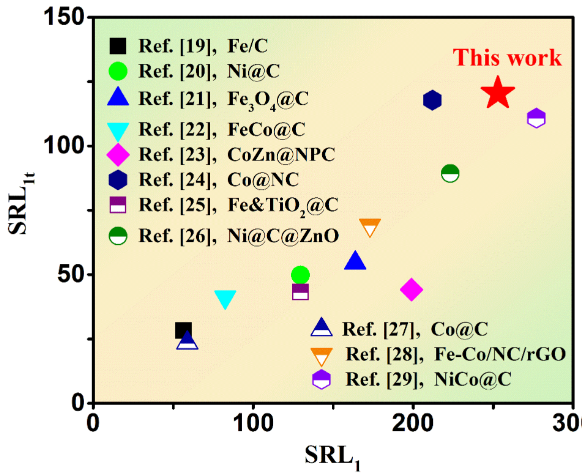

Fig. S16 The specific reflection loss of Co/Co_3O_4@HCNs compared with other reported MOFs derived counterparts

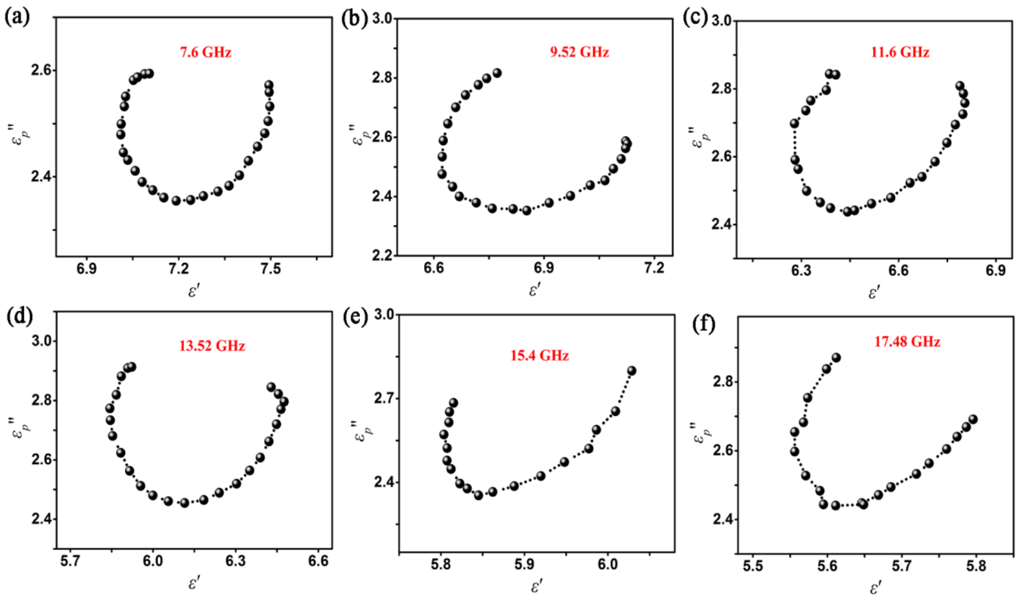

Fig. S17 Cole-Cole plots of Co/Co_3O_4@HCNs at 7.6 GHz **a**, 9.52 GHz **b**, 11.6 GHz **c**, 13.52 GHz **d**, 15.4 GHz **e** and 17.48 GHz **f**

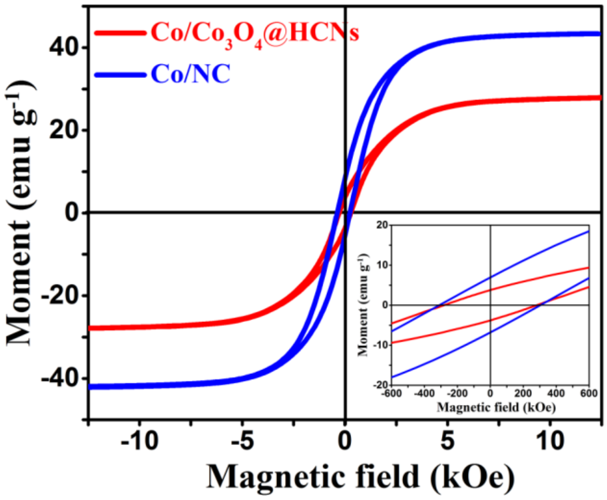

Fig. S18 The hysteresis loop of Co/NC and Co/Co_3O_4@HCNs

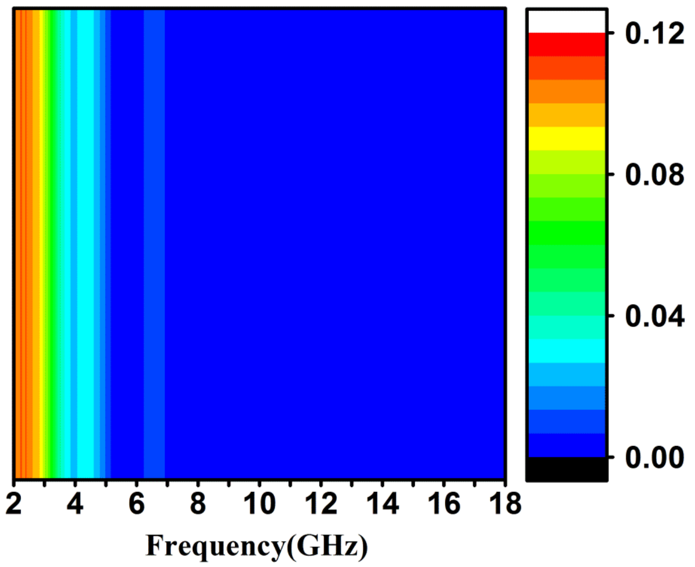

Fig. S19 The eddy current coefficient ($\mu''(\mu')^{-2}f^{-1}$) of Co/Co_3O_4@HCNs
